# Football players’ strength training method using image processing based on machine learning

**DOI:** 10.1371/journal.pone.0287433

**Published:** 2023-06-16

**Authors:** Xiaoxiang Cao, Xiaodong Zhao, Huan Tang, Nianchun Fan, Fateh Zereg

**Affiliations:** 1 School of Physical Education and Health, Hangzhou Normal University, Hangzhou, 310036, Zhejiang, China; 2 School of Physical Education, Chengdu University of Technology, Chengdu, 610059, Xichuang, China; 3 Chengdu Sport University, 610041, Chengdu city, Sichuan province, China; Universidade Estadual Paulista Julio de Mesquita Filho - Campus de Bauru, BRAZIL

## Abstract

This work addresses the declining physical fitness levels observed in both football players and the general population. The objective is to investigate the impact of functional strength training on the physical capabilities of football players and to develop a machine learning-based approach for posture recognition. A total of 116 adolescents aged 8 to 13 participating in football training are randomly assigned to either an experimental group (n = 60) or a control group (n = 56). Both groups underwent 24 training sessions, with the experimental group engaging in 15–20 minutes of functional strength training after each session. Machine learning techniques, specifically the backpropagation neural network (BPNN) in deep learning, are utilized to analyze the kicking actions of football players. Movement speed, sensitivity, and strength are employed as input vectors for the BPNN to compare the images of players’ movements, while the similarity between the kicking actions and standard movements served as the output result to enhance training efficiency. The experimental group’s kicking scores are compared to their pre-experiment scores, demonstrating a statistically significant improvement. Moreover, statistically significant differences are observed in the 5*25m shuttle running, throwing, and set kicking between the control and experimental groups. These findings highlight the significant enhancement in strength and sensitivity achieved through functional strength training in football players. The results contribute to the development of training programs for football players and the overall improvement of training efficiency.

## 1. Introduction

The popularity of competitive sports, particularly football, has grown in tandem with cultural and economic advancements [1,2]. Modern football is characterized by its aggressive nature, high volume, intensity, extended duration, and demanding physical and technical requirements. To excel in this sport, football players must undergo rigorous training to optimize their physical fitness and adaptability [3]. Physical fitness plays a crucial role in determining a player’s performance on the football field. However, Chinese football players tend to have lower overall physical fitness levels compared to their international counterparts [4,5]. This discrepancy can be attributed to the absence of scientific training plans and the focus on technical aspects rather than physical attributes, which is more prevalent in Asian countries. The decline in youth fitness levels in China is a concerning trend, prompting the government to implement measures aimed at improving the health and fitness of the general population [6,7]. It is important to tailor training approaches to different age groups. For example, children aged 7 to 9 should prioritize improving inter-muscular coordination through physical activities. Children aged 10 to 13 should focus on developing strength. Therefore, players require appropriate physical training methods to achieve maximum efficiency. Immersive glasses technology based on virtual reality has been developed by some researchers to enhance perception and improve athletes’ training outcomes [8]. This technology integrates cutting-edge information technologies such as human-computer interaction and virtual reality, providing a more immersive training experience. Functional training has gained significant attention from researchers worldwide, who have conducted extensive studies to explore its scientific foundations and efficacy.

This paragraph compiles several studies examining the effects of functional training on the strength and performance of football players. A study focused on the high-intensity tactical training program employed by the United States Marine Corps, which shares similarities with high-intensity functional training, and its growing popularity among military personnel. The authors also assessed the benefits and drawbacks of high-intensity functional training for the military [9]. A literature review has examined the effectiveness of high-intensity functional training in improving athletic performance [10]. The findings indicated that untrained and amateur participants experienced positive outcomes in terms of aerobic capacity, anaerobic performance, and muscle endurance. However, there were no significant changes observed in muscle strength and power. Functional training’s impact on postmenopausal women was examined in a 16-week study, which revealed significant improvements in body composition, functional adaptation, and lipid profiles [11]. Additionally, research has focused on the training of football players, considering the physical demands and tactics involved in the game, such as sprinting, stopping, turning, falling, jumping, and rushing. A study identified functional task deficits in soccer players experiencing chronic hip and groin pain [12]. Machine learning has been applied to athlete motion analysis, with one study successfully using hidden Markov patterns to accurately recognize badminton strokes [13]. This work highlighted the variations in acceleration among athletes executing identical stroke actions. Social network analysis combined with machine learning was employed to quantify individual player performance and team efficacy using football match data [14]. Video tracking techniques, specifically kernel correlation filtering, effectively monitored football player physical performance with minimal tracking errors and maintained frame rates [15]. Convolutional neural networks (CNNs) have been utilized to identify table tennis strokes, achieving an accuracy range of 85.0% to 95.0% [16]. Similarly, the backpropagation neural network (BPNN) algorithm successfully recognized different hand movements with an accuracy exceeding 95% [17]. The BPNN combined with Kinect technology was also employed to recognize human actions, demonstrating accurate recognition capabilities based on learned action features [18].

Despite the available research, there is a dearth of studies investigating the specific relationship between functional training and football players. Machine learning algorithms have demonstrated proficiency in recognizing human and athletic movements. While functional training has shown the potential to enhance athletes’ strength, there is a limited body of evidence specifically focused on the impact of functional training on the strength of football players. Furthermore, the imbalanced nature of sample data for feature extraction during changes in athletes’ actions presents a challenge in the recognition task. Further investigation is warranted in this area to provide a comprehensive understanding of the effects of functional training on the strength and performance of football players.

In this work, the movements of football players are identified using machine learning techniques to investigate the effectiveness of strength training in football. A total of 116 young participants aged 8 to 13, engaged in football team training, were randomly selected. Both groups undergo 24 training sessions, with the experimental group receiving an additional 15–20 minutes of functional strength training after each session. Machine learning algorithms are employed to recognize the distinct postures adopted by football players during training. Specifically, a BPNN is utilized to analyze the kicking motion of the players. By comparing images of the players’ movements using the BPNN, incorporating factors such as speed, sensitivity, and strength as input vectors, the output result measures the similarity between the players’ kicking action and the standard action. This approach aims to enhance training efficiency. Subsequently, the experimental scores of the kicking motions for both the experimental and control groups are compared against the standard movements. The findings of this work offer a valuable theoretical foundation for the development of training programs for football players, with the potential to enhance training efficiency and overall performance.

## 2. Method

### 2.1 Research subject

A total of 116 students within the age range of 8 to 13, who are actively engaged in football training, are selected as participants for this work. These students are randomly assigned to either the experimental group (consisting of 60 students) or the control group (comprising 56 students). Over the course of this work, both groups undergo a series of twenty-four training sessions, with each group following its own specific curriculum. Prior to participating in this work, the minors involve, along with their respective guardians, provided informed consent by signing a consent form, thereby indicating their willingness to take part in the experiment.

### 2.2 Research methods

1. Literature review: This work incorporates a comprehensive analysis of existing research to gain insights into the perspectives of other scholars regarding machine learning for recognizing athletes’ movements. By examining previous studies, this work aims to establish a theoretical foundation and evaluate the reliability and validity of this work.
2. Expert interview: The design of student courses is based on a scientific approach that involves input from both football coaches and experts in functional strength training. By incorporating the knowledge and expertise of these professionals, the course design ensures that the selected indicators are representative of the overall training process.
3. Experiment: The participants in the experimental group engage in functional strength training sessions following each regular course, while the control group does not receive such training. This experimental setup enables the investigation of whether functional strength training has a positive impact on the development of speed and strength in football players. By comparing the outcomes between the two groups, valuable insights can be obtained regarding the benefits of functional strength training.

### 2.3 Content of functional strength training

Functional strength training is a training approach that aims to improve the overall muscular contraction strength and efficiency of individuals, taking into account their specific characteristics and needs. Unlike traditional training methods that focus on isolated muscle development for specific movements, functional strength training emphasizes a balanced approach. It encompasses a range of exercises that target opposing movements such as pushing and pulling, as well as exercises that address different areas of the body, such as the hips and knees. The primary goal of functional strength training is to enhance the ability to engage a greater number of muscle fibers rather than solely focusing on muscle hypertrophy. This approach leverages the body’s elasticity to generate explosive power. By promoting the activation of multiple muscle groups and optimizing neural coordination, functional strength training contributes to the improvement of maximum strength and power output. The concept of functional training seeks to establish a standardized training method that can benefit athletes across various sports. It recognizes the importance of the body’s kinematic chain and aims to enhance overall coordination and functional performance. Through the incorporation of functional strength training, individuals can unlock their full potential and achieve improved physical capabilities in sports and other activities of daily life.

The functional strength training program utilized here includes the following exercises:

1. Supine leg lifting with feet clamping a football:

This exercise aims to target and strengthen the rectus abdominis and iliopsoas muscles. Participants lie on their backs, gripping a football with their feet to prevent it from rolling away. The exercise involves lifting the leg quickly and lowering it slowly while simultaneously lifting the hands and head off the ground. Each round consists of two sets, with each set comprising ten repetitions of leg lifts and drops.

2. Supine hip turning with knees clamping a football:

This drill focuses on enhancing the hip flexors and iliopsoas muscles. Participants lie on their back with arms extended to the sides. They bend their knees to a 90-degree angle while gripping a solid ball between their knees. The exercise involves twisting the hips from left to right while maintaining steady breathing and engaging the abdominal muscles. Each participant performs two sets of 15 repetitions for each movement.

3. Single-sided plank:

The aim of this exercise is to target multiple muscle groups, including the latissimus dorsi, erector spinae, gluteus maximus, deltoid muscle, rectus abdominis, and abdominal oblique muscles. Participants start in a prone position and bend their left elbow joint to support their body weight, while simultaneously raising their right arm forward. During the training, the participants straighten their left leg to support the ground and lift the front of their right foot. It is important to maintain steady breathing throughout the entire training process, keep the back tightened, and avoid any shaking of the raised arm and leg. Each set consists of 15 repetitions, and participants perform two sets of training.

4. Single leg squat:

The objective of this exercise is to target the hip muscles and quadriceps of the players. Participants begin by assuming a standing posture with their weight on the right foot, maintaining balance. They then extend the left leg backward, placing the right hand behind the ears. The exercise involves squatting down, touching the right foot with the left hand, and standing back up. It is crucial to avoid bending over during the squatting movement. Participants should perform two sets of training, with each set comprising 15 repetitions.

5. Supine leg swinging with feet clamping a football:

The purpose of this exercise is to target the players’ rectus abdominis and iliopsoas muscles. Participants lie on their backs with their feet clamped around a football to prevent it from dropping during training. Using the waist as the axis, they swing their legs from left to right, with their hands placed on both sides of the body to maintain balance. The upward swing should be swift, while the downward swing should be done leisurely. Each set consists of 10 repetitions, and participants perform two sets of training.

6. Supine head leg crunches with feet clamping a football:

This exercise focuses on exercising the players’ rectus abdominis and iliopsoas muscles. Participants lie on their backs with their feet pinned to the football, lifting their legs, curling their abdominals, and raising their hands. They slightly raise their head and lift their legs straight off the ground. Each set comprises 15 repetitions, and participants perform two sets of training.

These exercises have been incorporated into the functional strength training program to specifically target and strengthen the rectus abdominis and iliopsoas muscles of the football players. Participants can enhance their core stability, abdominal strength, and overall lower body muscular endurance by including supine leg swinging and supine head leg crunches with feet clamping a football.

### 2.4 Experimental teaching methods

Over the course of one month, specifically from July 2019 to August 2019, the experimental group underwent regular training sessions from 9 a.m. to 11 a.m. daily, while the control group attended scheduled classes from 5 p.m. to 7 p.m. each day. Both groups implemented game-based learning strategies to ignite the students’ enthusiasm for football. The training primarily focused on application drills such as passing and dribbling, aiming to enhance the players’ skills in these areas.

Following each training session, the experimental group participated in 15–20 minutes of functional strength training. Throughout the entire project, three coaches were involved, ensuring the elimination of potential instructor bias. There were no changes made to the training aids used by either the experimental or control groups during the duration of this work.

Data were collected and analyzed using a machine learning-based posture identification technique, focusing on ten football players and their seven postures: inaction, walking, running, juggling, kicking, catching, and lifting the ball. Each motion was recorded fifty times, resulting in a total of 5000 samples. The participants assumed standard football positions based on their typical physical activity levels during the data collection process.

### 2.5 Test indicator selection

The indicators examined in this work encompassed the player’s speed, sensitivity, and strength. To assess speed, the 10-meter and 30-meter sprints were employed as performance measures. Sensitivity was evaluated using the Illinois agility test and the 5*25-meter shuttle run. Lastly, strength was evaluated through throws and set kicking drills, which provided a measure of the players’ overall strength capabilities.

### 2.6 Football players’ posture recognition using machine learning

Data on acceleration and angular velocity were collected from football players’ positions. $a_{n}^{x}$, $a_{n}^{y}$, and $a_{n}^{z}$ represent the *n*-th sampling point’s acceleration on the *x*, *y*, and *z* axes. $g_{n}^{x}$, $g_{n}^{y}$, and $g_{n}^{z}$ represent the *n*-th sampling point’s angular velocities on the *x*, *y*, and *z* axes. Eq (1) shows the acceleration’s vector sum at the *n*-th point.


$$a_{n}=\sqrt{{\left(a_{n}^{x}\right)}^{2}+{\left(a_{n}^{y}\right)}^{2}+{\left(a_{n}^{z}\right)}^{2}}$$ (1)

Similarly, Eq (2) shows the angular velocity’s vector sum at the *n*-th point.


$$g_{n}=\sqrt{{\left(g_{n}^{x}\right)}^{2}+{\left(g_{n}^{y}\right)}^{2}+{\left(g_{n}^{z}\right)}^{2}}$$ (2)

The data acquisition module captures three acceleration vectors and three angular velocity vectors. The vector sum of acceleration and angular velocity is computed using Eqs (1) and (2), resulting in a total of eight vectors. These eight vectors are then combined to form a single 8-dimensional feature matrix. During data collection, *N* sampling points are gathered, with each sample representing an *N*×8 feature matrix. The mean and variance are utilized as time-domain features for gesture recognition in football players. The following quotation presents the mean values for each sample point.


$$\mu_{a}=\frac{1}{N}\sum_{n=1}^{N}a_{n}$$ (3)

Eq (4) shows each sample point’s variance.


$$\delta^{2}=\frac{1}{N}\sum_{n=1}^{N}{\left(a_{n}-\mu_{a}\right)}^{2}$$ (4)

The time-domain features extracted comprise a total of 16 dimensions of attitude parameters. These include:

Acceleration sensors: x-axis, y-axis, z-axis, and the mean value of the acceleration vector sum.

Angular velocity vector sensors: x-axis, y-axis, z-axis, and mean value of the angular velocity vector sum.

Acceleration sensors: x-axis, y-axis, z-axis, and variance of the acceleration vector sum.

Angular velocity vector sensors: x-axis, y-axis, z-axis, and variance of the angular velocity vector sum.

These 16 dimensions provide valuable information about the attitude and movement characteristics of football players.

Following feature extraction, a 32-dimensional feature parameter set is generated for football players’ posture recognition. However, to improve the recognition performance and efficiency of the classifier, it is important to filter out irrelevant or redundant feature parameters that are closely related to the football player’s hand gestures. This can be achieved by selecting relevant features to reduce the dimensionality of the data. In this work, the principal component analysis (PCA) method was employed for feature parameter selection after conducting experimental tests. PCA helps identify the most informative features and reduce the dimensionality of the dataset, thereby enhancing the accuracy and efficiency of football hand gesture recognition.

### 2.7 Acquisition of ball-kicking data of players

**Fig 1:**
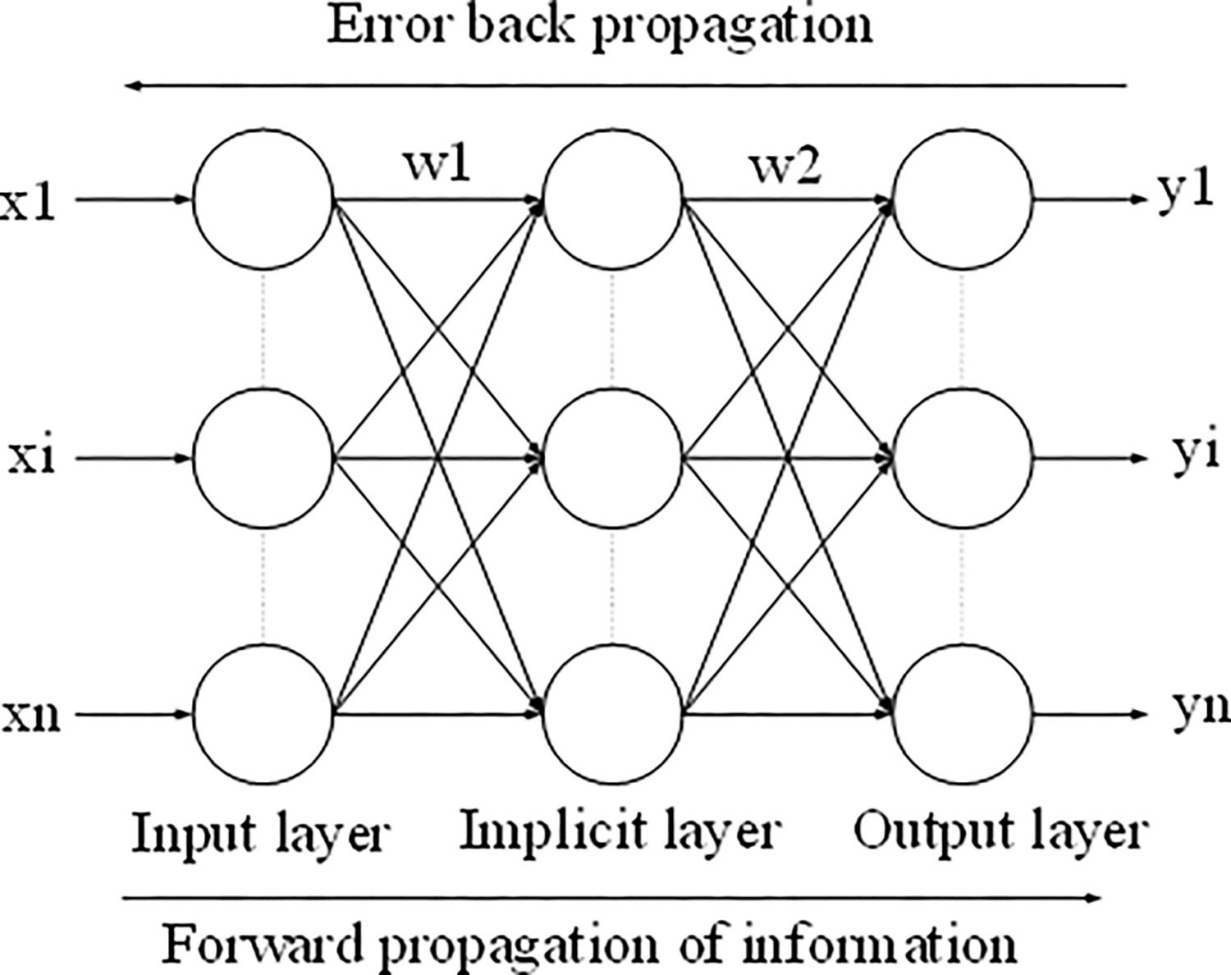
BPNN structure.

The deep learning (DL) method aligns with the concept of an artificial neural network (ANN), which serves as a machine learning architecture. The neural network, functioning as an algorithm, trains the weights connecting individual units within the network. Drawing inspiration from the workings of the human brain, ANN algorithms have the capacity to learn and adapt to new scenarios. In the human brain, input signals are received and processed through the nervous system, while external stimuli are sensed through neurons that convert electrical signals from nerve endings. DL-based neural networks are mathematical models that emulate the neural system of the human brain. These networks exhibit high fault tolerance, fast learning and self-adaptation rates, and the ability to approximate nonlinear functions. They can be effectively employed for tasks such as binary image recognition, prediction, and fuzzy control of binary images. The BPNN is an example of a three-layer feedforward neural network comprising the input, hidden, and output layers. The structure of the BPNN is illustrated in Fig 1.

**Fig 2:**
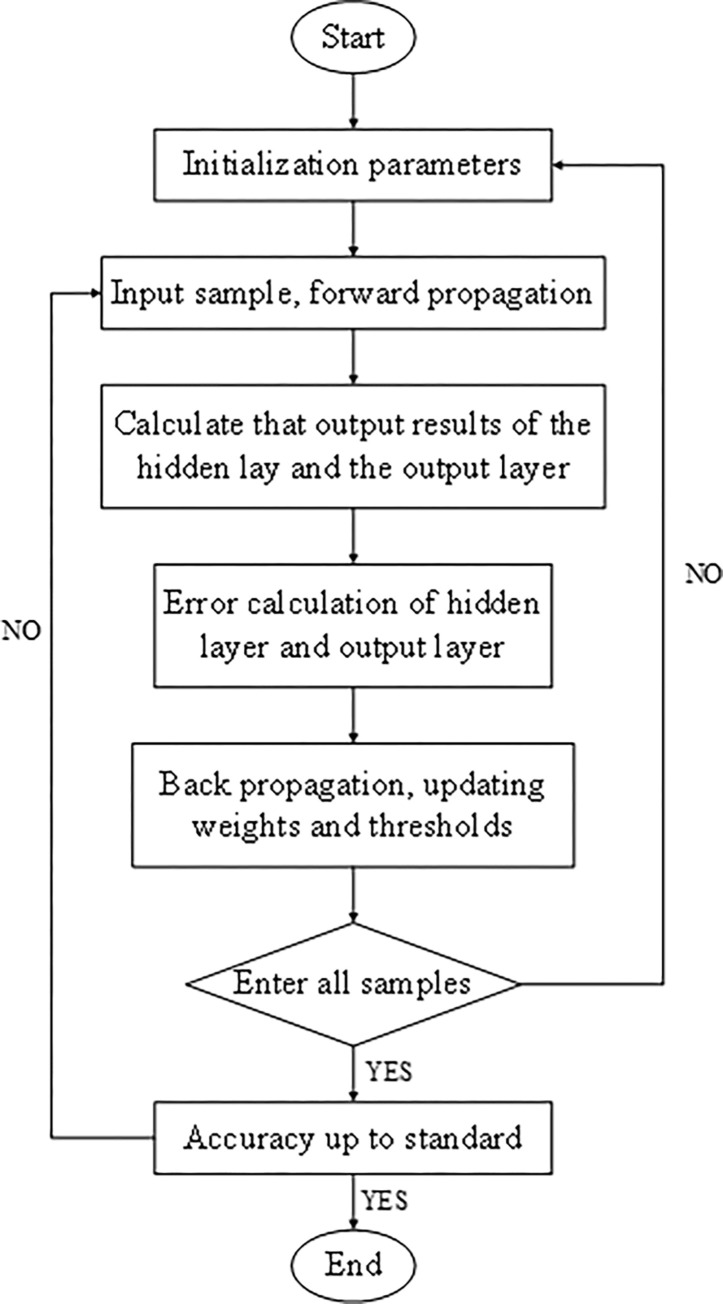
Algorithm process of BPNN.

In the BPNN, each layer consists of n neurons, and there are connections between each layer. However, the neurons within a single layer are not interconnected. The BPNN algorithm involves two main processes: error backpropagation and forward information propagation [19]. The detailed steps of these processes are depicted in Fig 2.

In the forward propagation process, the input object is divided into n input vectors. The weight coefficient is denoted as “*w*” and the bias vector as “*b*”. The input vector “*x*” undergoes a linear operation, as shown in Eq (5).


$$z_{i}=\sum_{i}w_{i}{}_{n}•x_{n}+b_{i}$$ (5)

In Eq (5), “*z*_*i*_” represents the output of the *i*-th neuron. The network performs calculations layer by layer, starting from the input layer until reaching the output result. In BPNN, the sigmoid function is commonly used as the activation function for the neurons in the hidden layers to enhance the algorithm’s expressive ability. Eq (6) represents the sigmoid function “*g*(*z*_*i*_)”.


$$g(z_{i})=\frac{1}{1+e^{-z_{i}}}$$ (6)

The domains of the mentioned functions are sets of real numbers, and their range is [0, 1]. After obtaining the outputs from all the hidden layers, the cross-entropy loss function is used to measure the discrepancy between the predicted values and the observed values, thus evaluating the predictive capability of the model. The loss function can be represented by Eq (7).


$$loss=-\frac{1}{n}\sum_{x}[y\ln g(z_{i})+(1-y)ln(1-g(z_{i}))]$$ (7)

In Eq (7), the variable *y* stands for the actual output.

During the error backpropagation process, the gradient of the loss function is propagated backward from the output layer to the hidden layer, and the loss value is distributed to each layer of neurons. Through continuous iteration, the parameters between layers are updated to minimize the error between the actual output and the expected output values. This procedure enhances the robustness of the BPNN by adjusting the weights and thresholds associated with the smallest error [20]. According to the gradient descent method based on the loss function, the largest gradient descent occurs along the backpropagation process.


$$w_{i+1}=w_{i}-lr•\nabla$$ (8)

$$\nabla =\frac{\partial loss}{\partial w}$$ (9)

In Eqs (8) and (9), *w*_*i*+1_ represents the updated weight coefficient, *w*_*i*_ refers to the current weight coefficient, *lr* signifies the learning rate for each iteration, and ∇ represents the gradient of the loss function.

The selection of the number of nodes in the hidden layer is a challenging aspect of feature layer fusion based on BPNN. Currently, there is no standardized method for determining the number of nodes in the hidden layer, which directly affects the overall performance of the network. Therefore, careful consideration must be given to the selection of hidden layer nodes [21,22]. The concept of a “suitable amount” should be applied when choosing the number of hidden layer nodes. Having an excessive number of nodes in the BPNN may lead to longer training times and may not achieve the desired learning outcome. On the other hand, having too few nodes results in an overly simplistic BPNN structure with lower recognition accuracy, robustness, and interference resistance [23,24]. The number of hidden layers can be determined using Eq (10).


$$N_{h}=\sqrt{N_{1}+N_{0}}+L$$ (10)

In Eq (10), *N*_*h*_ stands for the desired number of hidden layer nodes, *N*_*1*_ signifies the number of input nodes, *N*_*0*_ represents the number of output nodes, and *L* refers to an integer between 1 and 10. Based on practical training experience, *L* is typically chosen as a large value, generally greater than half of the sum of the input and output nodes.

For this work, a dataset was collected by inviting twenty male football players who had different heights and weights to perform kicking actions. Among the players, one player stood out as being taller than the rest, and his kicking action data was used as the standard action. High-speed cameras were utilized to capture the kicking action data, with a frame rate of 25fps and a resolution of 1024×1280. The BPNN algorithm was employed to analyze the similarity between the kicking actions of young players and the standard action, providing valuable insights for their football training.

### 2.8 Evaluation criteria for similarity of image features

The calculation of image feature similarity plays a crucial role in the accuracy of subsequent image retrieval. Different similarity measurement functions can yield diverse image retrieval results. In this work, the following similarity measurement function is utilized:

(1) Euclidean Distance: Euclidean Distance is a widely used distance metric for comparing two points in three or more dimensions of space [25]. It is a straightforward approach for quantifying the similarity between scenes. A smaller Euclidean distance indicates a greater similarity, while a larger Euclidean distance suggests a lower similarity.

$$d(x,y)=\sqrt{\sum_{i=1}^{n}{\left(x_{i}-y_{i}\right)}^{2}}$$ (11)

In Eq (11), *x*_*i*_ and *y*_*i*_ represent the feature vectors of image *x* and image *y*, respectively, and *n* represents the dimension of the image feature vector.

(2) Cosine Similarity: Cosine similarity utilizes the cosine value of the angle between two vectors in the vector space to quantify their dissimilarity [26]. The closer the cosine value is to 1, the smaller the angle between the vectors, indicating a higher degree of similarity between the two individuals.

$$d(x,y)=\frac{\sum_{i=1}^{n}x_{i}y_{i}}{\sqrt{\sum_{i=1}^{n}x_{i}}\sqrt{\sum_{i=1}^{n}y_{i}}}$$ (12)

In football players’ joint point detection, a CNN is used to convolve the input images and generate confidence maps, also known as hot spot maps, for detecting the joint points. Multi-stage learning is employed, where each stage refines the learning and detection results of the previous stage, aiming to bring the final results closer to the target values. Let $S_{jk}^{*}(p)$ represent the confidence map of the *k*-th athlete, and *x*_*jk*_ denote the actual value of the *j*-th joint point for this athlete. The predicted value at point *P* can be expressed using the following Gaussian function:

$$S_{jk}^{*}(p)=\exp-\frac{{\left\|p-x_{jk}^{}\right\|}_{2}^{2}}{\sigma^{2}}$$ (13)

After detecting the joint points, it is necessary to connect them to form a complete human body model. However, if the connection is solely based on the confidence of the detection points, errors may occur when multiple points are involved. A local affinity domain method incorporates limb direction information into the predictions to address this issue, generating a two-dimensional (2D) vector code. Typically, the human body model can be represented as a simple connection of multiple joint points. After polynomial fitting of the body joint points in the x-axis and y-axis directions, the coefficients of the polynomial fitting are used as features for data analysis. PCA is then applied to perform data dimension reduction. PCA aims to reduce the dimensionality of the data by utilizing the eigenvectors of the sample covariance matrix. It constructs an orthogonal transformation that minimizes the mean square error (MSE). Through this transformation, the characteristics of the samples are decomposed into orthogonal components, revealing the fundamental components of the data information.

In the data training phase, several steps are followed to prepare the data for classification using Support Vector Machines (SVMs). First, the average of the training data samples is calculated, and this average is subtracted from each training data point. Next, the covariance matrix and eigenvalues are computed. The dimension of the matrix is then reduced based on the contribution rate of the calculated eigenvectors. This reduction helps to eliminate redundant or less informative dimensions. Finally, the final training data is obtained by multiplying the difference between each training sample and the average data by the dimensionality reduction matrix. During the data testing phase, a similar process is applied. The average of the training data is subtracted from each testing data point, and the resulting difference is multiplied by the dimensionality reduction matrix.

Once the data is prepared, it is fed into SVMs for classification and action recognition. SVMs are capable of handling nonlinear problems by using a kernel function to map the data into a higher-dimensional feature space (Hilbert space) where the problem becomes linearly separable. In this case, the Gaussian kernel function is typically used to transform the feature space, allowing the SVM training model to successfully recognize different actions.

### 2.9 Statistical methods

This work utilizes the Berkeley Multi-Channel Human Action Detection (MHAD) dataset to evaluate the effectiveness of the BPNN algorithm for action recognition. The MHAD dataset includes reference background and depth images, which are used to extract the first-layer feature vectors. The dataset consists of 11 different movements performed by a group of seven men and five women. Each movement is repeated five times, resulting in a total of 660 action sequences. The recording duration for the dataset is approximately 82 minutes. This dataset provides a valuable resource for training and evaluating the BPNN algorithm’s performance in action recognition tasks.

The player training data is analyzed using IBM’s statistical software SPSS 26.0 (Statistical Product and Service Solutions 26.0), developed by IBM Corporation in New York, USA. In the analysis of the players’ basic situation, the data were presented using a percentile system. A single-factor analysis was conducted to examine the differences between different situations. In statistical analysis, a commonly used threshold for determining statistical significance is a p-value of less than 0.05. If the calculated p-value is less than 0.05, it indicates that the observed difference between the groups is statistically significant. In other words, there is a significant difference between the situations being compared.

## 3. Results

This section presents a comprehensive analysis of experiments conducted to validate the effectiveness of image processing technology based on DL in guiding football players during functional strength training. Each experiment result is thoroughly examined and discussed. The following subsections provide a clear overview of the findings:

Section 3.1 presents the accuracy results of the BPNN algorithm for recognizing single movements in the MHAD dataset.

Section 3.2 analyzes the statistical differences between the experimental and control football players in terms of movement speed, sensitivity, and strength test metrics. The focus is on highlighting the improvements observed in each testing metric within the experimental group.

Section 3.3 compares the performance of the experimental and control football players in 10-meter sprints, 30-meter sprints, and fixed-point kicks. It examines and discusses the observed differences between the two groups.

Section 3.4 illustrates the performance test results of the experimental hidden node within the BPNN framework. This analysis aims to evaluate the effectiveness and capabilities of the hidden node within the experimental setup.

Section 3.5 provides a detailed analysis of the performance test results of the BPNN model. This examination sheds light on the model’s performance and effectiveness in the context of the experiments.

Section 3.6 presents the performance test results of the experimental hidden node and highlights any observed differences in functional strength training between the experimental and control groups.

### 3.1 Posture recognition results using machine learning and BPNN

Table 1 displays the accuracy results of the BPNN algorithm for single-action recognition in the MHAD dataset. It is essential to assess data quality by examining the efficiency of records with varying lengths since data quality is directly related to the length of the record. Actions with longer recordings, such as “sit and stand” or “bend to raise hand” in the MHAD dataset, exhibit better performance compared to actions with shorter recordings. It is worth noting that the performance of the BPNN technique can be significantly enhanced by increasing the utilization of unlabeled data as input to the deep network. This characteristic makes the algorithm well-suited for practical applications where unlabeled data is abundant.

**Table 1:** Comparison of single-action recognition accuracy rate in MHAD dataset.

Action	Length	Performance
Jump	5	70.6
Jumping firecrackers	7	93.0
Bend to raise the hand	12	90.5
Boxing	10	83.4
Two-hands wave	7	95.0
Right-hand wave	7	88.5
Applause	5	84.5
Throwing the ball	3	47.5
Sit and stand	15	95.6
Sit	2	32.7
Stand	2	28.8
Average accuracy	-	85.8

During the collection of sport posture data, participants perform specific football actions based on their exercise habits. These football postures consist of distinct upper limb actions and lower limb actions, which are recognized separately. As a result, separate classifiers are constructed for upper limb actions and lower limb actions. The recognition results of random forest classifiers, support vector machines, and Bayesian networks for upper and lower limb actions are presented in Table 2.

**Table 2:** The recognition results of classifiers of random forest, support vector machine, and Bayesian network.

Posture	Random forest	Support Vector Machine	Bayesian network
Walking	96.5	94.2	91.0
Running	98.2	94.8	92.1
Juggling	93.2	95.3	91.7
Kicking	94.4	95.6	92.8
Catching	90.2	97.1	90.5
Lifting	91.4	91.8	90.8

### 3.2 Comparison of the results in the experimental group before and after the experiment

**Fig 3:**
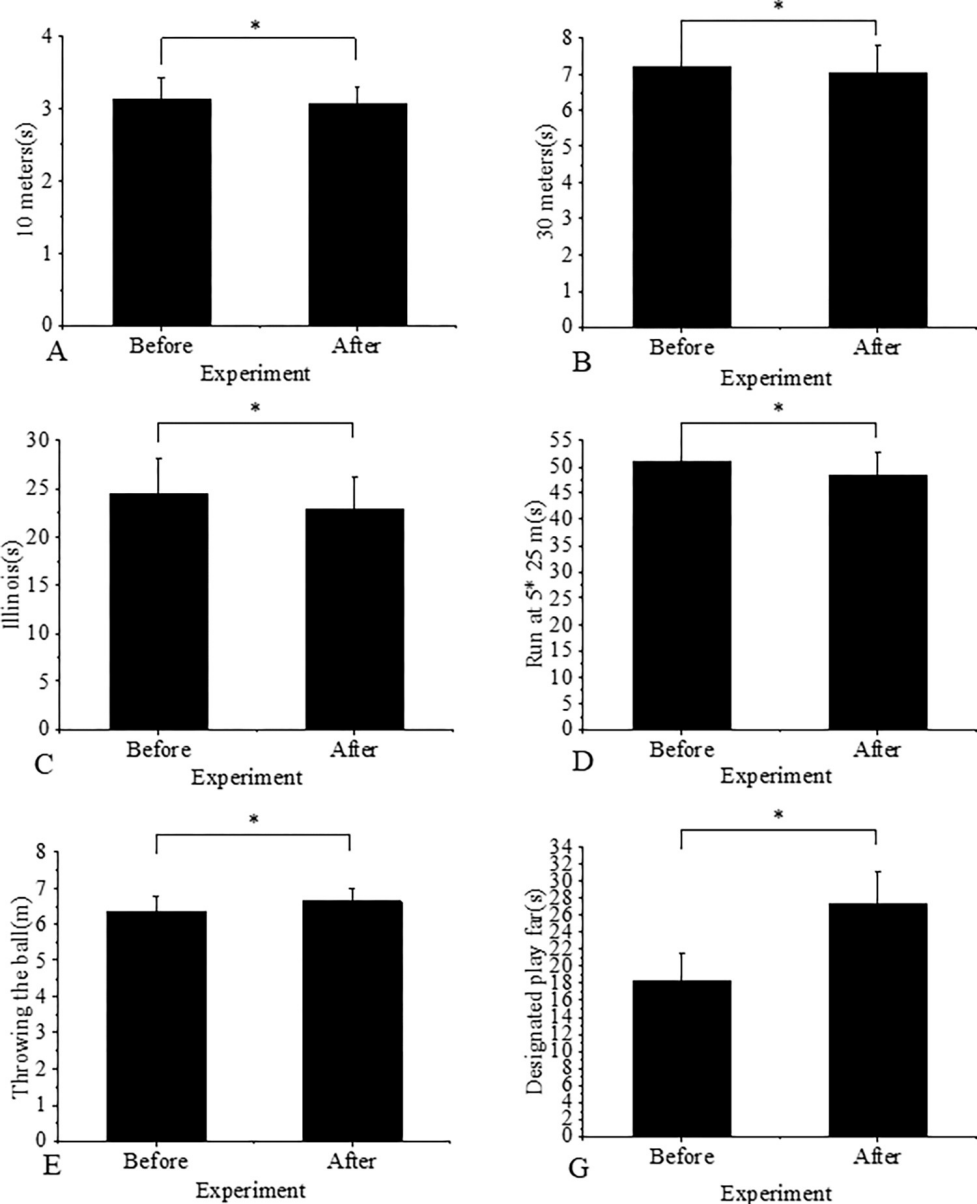
Comparison of the results in the experimental group before and after the experiment (Note: * indicates that the difference is statistically significant).

Fig 3 depicts the pre- and post-experiment results in the experimental group.

The experimental group demonstrated significant improvements in various performance metrics after the experiment. Specifically, there was a substantial 1.6% improvement (P = 0.000 < 0.05) in the 10-meter running performance. The 30-meter run performance showed a 1.95% improvement (P = 0.000 < 0.05), and the Illinois run performance increased by 6.38% (P = 0.000 < 0.05). Additionally, the experimental group exhibited a 5.29% improvement (P = 0.000 < 0.05) in the 5x25m shuttle running performance and a 4.73% improvement (P = 0.000 < 0.05) in throwing performance. Notably, the kicking performance of the experimental group significantly increased by 49.73% (P = 0.000 < 0.05) as a result of the trial.

### 3.3 Comparison of the results between the experimental and control groups

**Fig 4:**
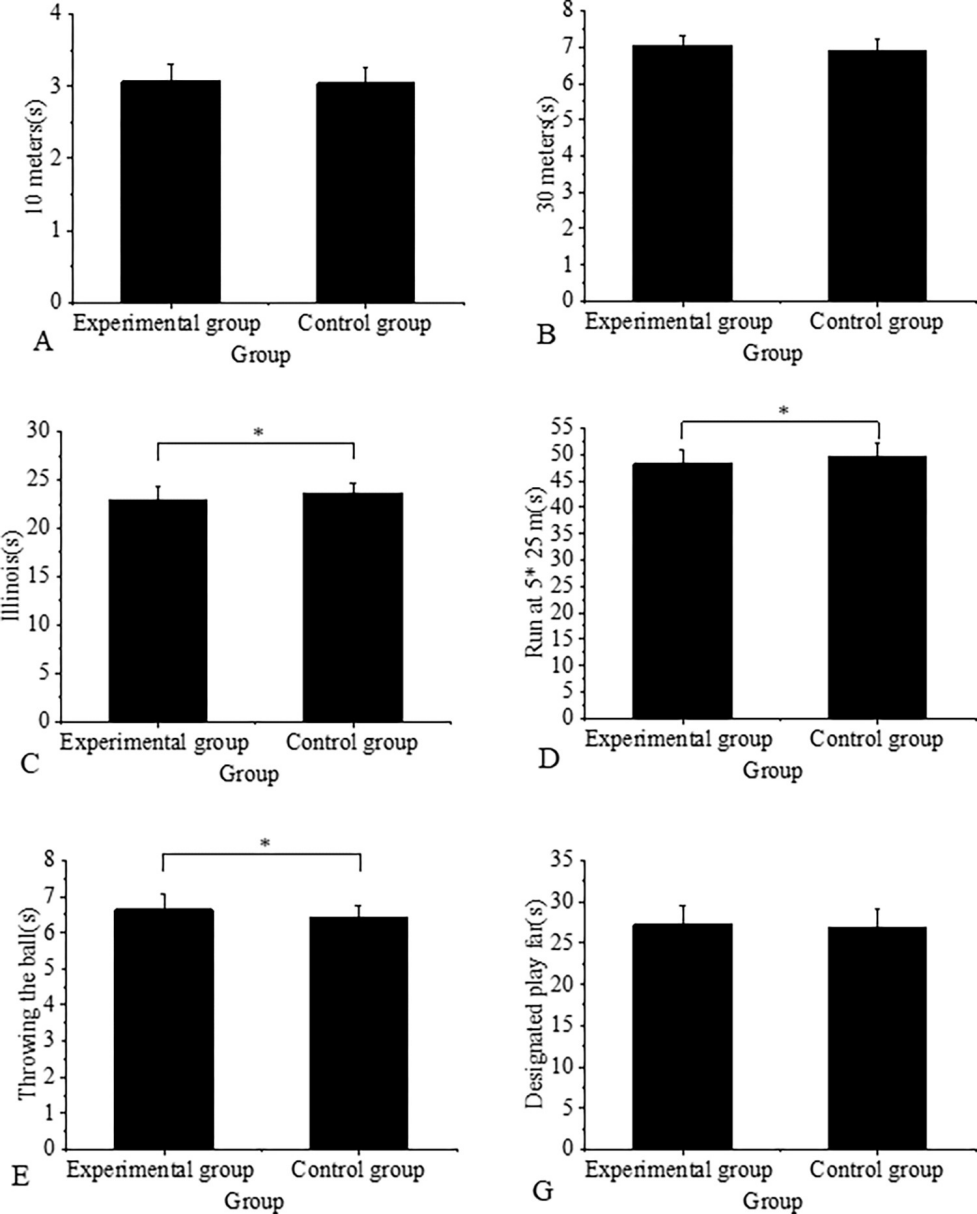
Comparison of the results between the experimental and control groups(Note: * Indicates a significant difference).

Fig 4 compares the results between the experimental and control groups.

There were no statistically significant differences (P > 0.05) observed in the performances of players who underwent functional strength training compared to those who did not, specifically in the 10-meter running, 30-meter running, and set kicking tests. The experimental group achieved an average score of 3.07 ± 0.23 in the 10-meter running test, while the control group scored 3.04 ± 0.23, and this difference was not statistically significant (P = 0.659). Similarly, in the 30-meter running test, the experimental group achieved a score of 7.05 ± 0.25, while the control group scored 6.90 ± 0.31, resulting in a non-statistically significant difference (P = 0.060). However, there was a statistically significant difference (P = 0.031) between the experimental group’s performance (22.88 ± 1.42) and the control group’s performance (23.63 ± 1.08) in the Illinois running tests. The 5x25 shuttle running tests also revealed a statistically significant difference (P = 0.033) between the experimental group’s performance (48.30 ± 2.56) and the control group’s performance (49.75 ± 2.37). Similarly, a statistically significant difference (P = 0.038) was found between the experimental group’s throwing test performance (6.64 ± 0.43) and the control group’s performance (6.42 ± 0.33). However, the difference in mean scores between the experimental and control groups in the overall test scores was not statistically significant (27.28 ± 2.28 and 26.82 ± 2.27, respectively), with a P value of 0.461.

### 3.4 Test results of hidden layer nodes

Table 3 lists the test results of hidden layer nodes after multiple experiments based on Eq (6).

**Table 3:** Performance test results of hidden nodes of the BPNN algorithm.

Number of hidden layer nodes	The training steps	Maximum error	Best Validation Performance
6	139	0.0014	0.001301
7	128	0.0013	0.000501
8	127	0.0019	0.002715
9	61	0.0301	0.003502
10	135	0.0045	0.002013
11	134	0.0021	0.003015
12	55	0.0150	0.012131

Table 3 demonstrates that the performance of the BPNN remains consistent and the error is minimized when it has 7 hidden nodes. Therefore, the optimal number of hidden nodes for the BPNN is determined to be seven.

### 3.5 BPNN simulation results

**Fig 5:**
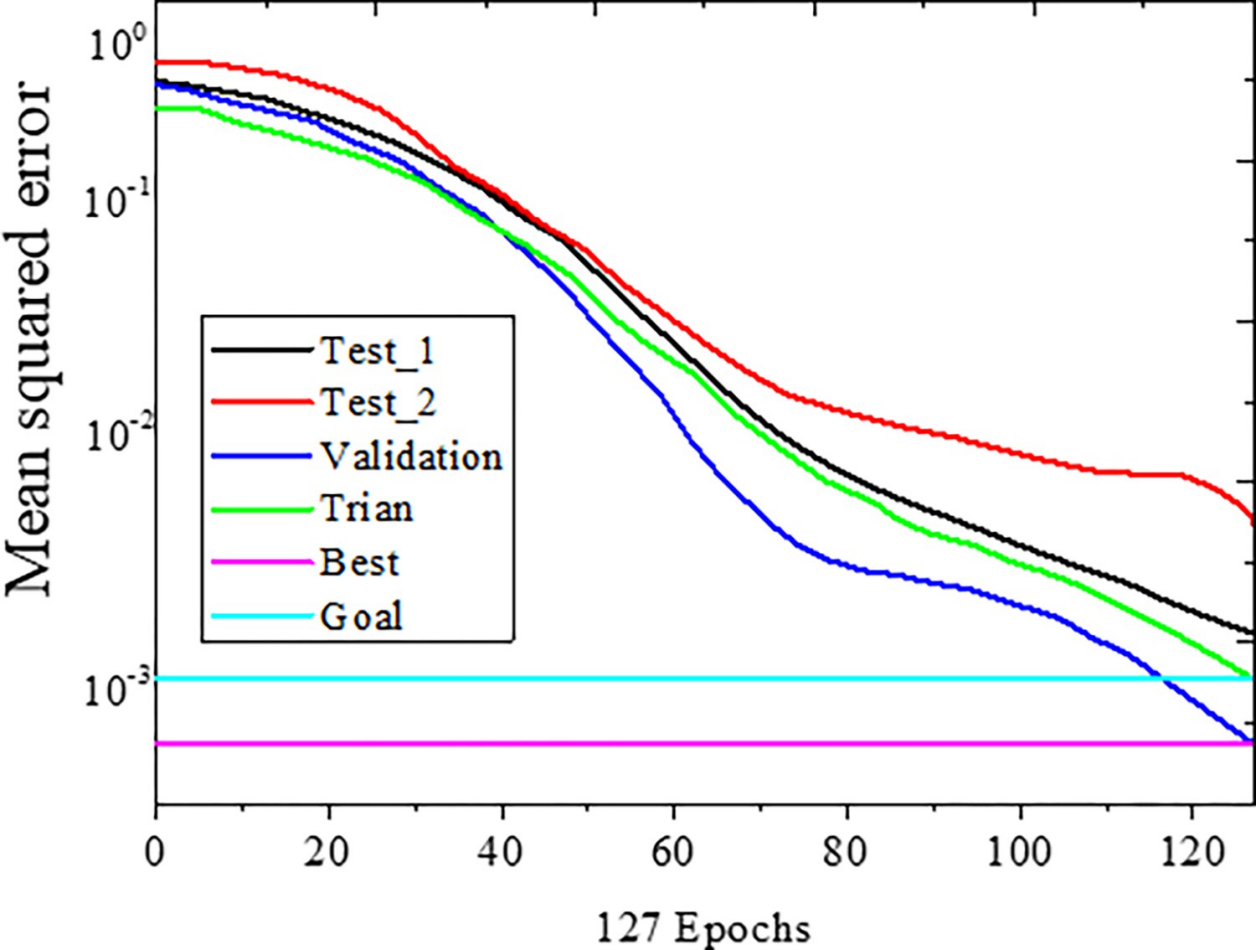
Simulation results of BPNN.

Fig 5 presents the simulation results of BPNN.

After 127 iterations, as depicted in Fig 5, the error of the BPNN reaches its lowest target value, indicating the excellent accuracy achieved by the BPNN. The trained BPNN exhibits a running time of 0.0276s and an MSE of 0.0013. A smaller MSE value corresponds to higher accuracy of the BPNN, thereby satisfying the high precision requirement.

### 3.6 Accuracy of kicking actions of football players

**Fig 6:**
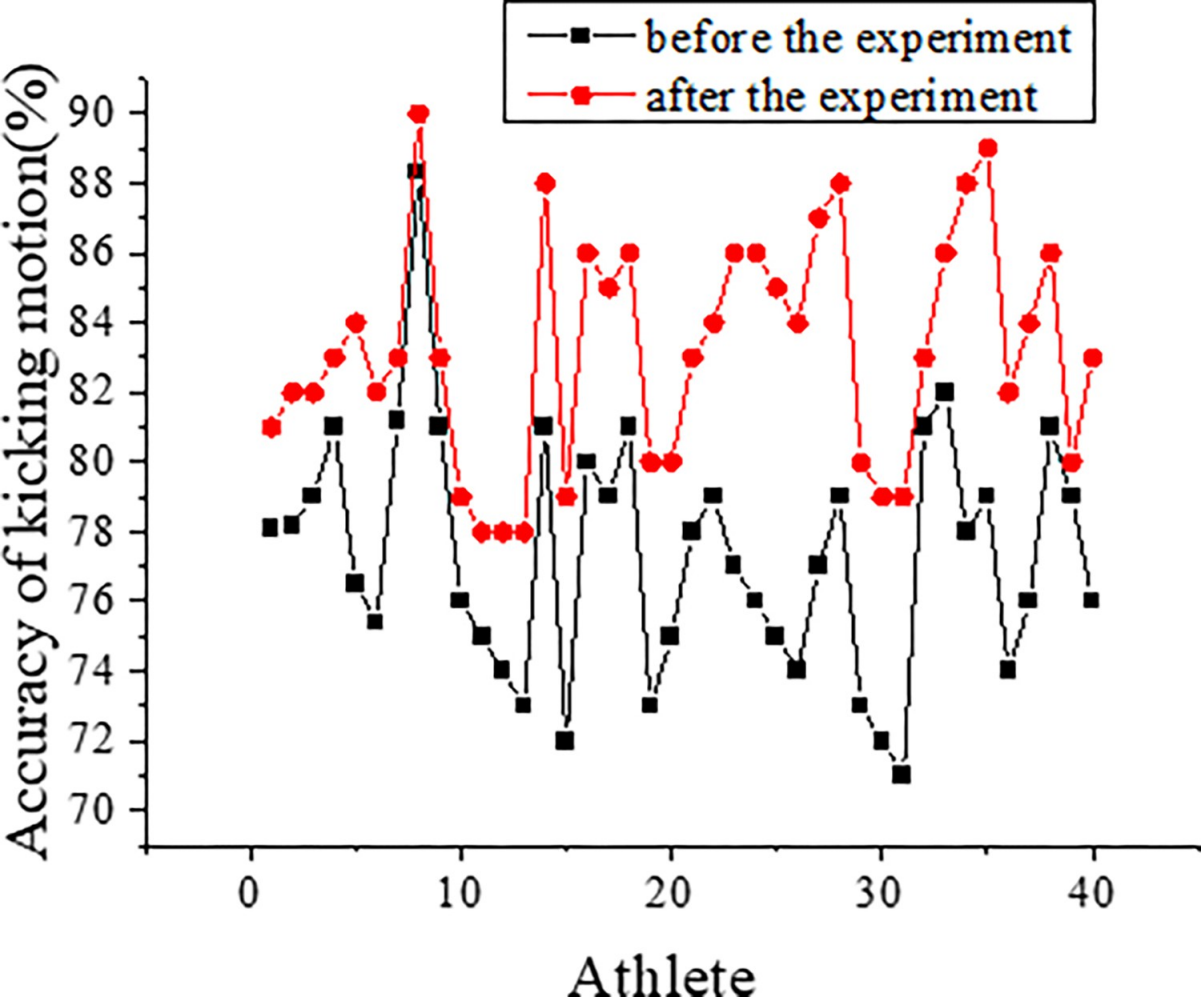
The accuracy of kicking actions of players in the experimental group.

**Fig 7:**
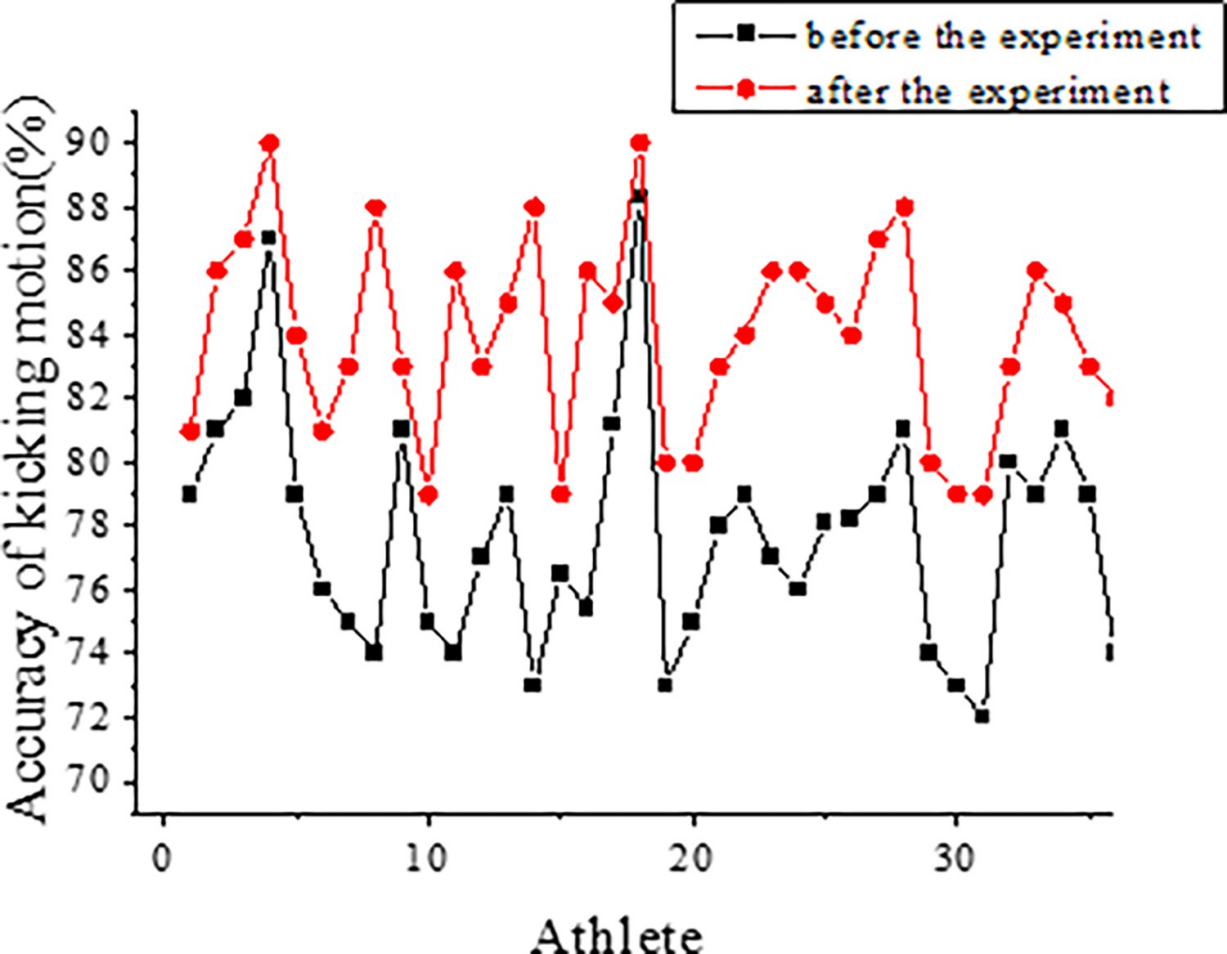
The accuracy of kicking actions of players in the control group.

Figs 6 and 7 present the results obtained from the analysis of 2D images of football players during kicking actions. The accuracy of all players’ kicking data is evaluated, and the findings are depicted in these figures.

There are no significant differences observed between the experimental and control groups before and after the experiment, as both groups received similar action training. Prior to the experiment, the action accuracy of the players in the experimental group was 73.2%, while the control group achieved an accuracy of 74.3%. After the experiment, the experimental group demonstrated an accuracy of 83.4%, while the control group showed an accuracy of 84.1%.

## 4. Discussion

The declining physical fitness of football players is a concerning trend in the current landscape. In light of this, the present work aims to investigate the impact of functional strength training on the physical capabilities of football players. By exploring the relationship between functional strength training and the physical fitness of football players, this research seeks to shed light on potential strategies for improving and maintaining their physical performance levels.

The experimental findings provide evidence that functional strength training is effective in enhancing the strength and sensitivity of football players. The observed improvements in strength can be attributed to the training’s ability to target and strengthen the players’ core muscles. Functional strength training stimulates the core muscles, particularly when there are imbalances in other body parts, resulting in improved utilization of athletic abilities and increased core strength during exercises. This, in turn, contributes to improved overall performance. However, the immediate impact on speed is not as evident. This could be due to the fact that speed performance improvement is closely tied to specific speed training, which may not have been explicitly included in the functional strength training program. Both the experimental and control groups likely had similar speed training components, resulting in comparable speed outcomes after the experiment. It is worth noting that the athletes may have already possessed a higher level of speed quality prior to the experiment, which could explain the lack of significant differences in speed improvement between the two groups. Previous research has examined the effects of functional strength training in various contexts. For example, a study focusing on weightlifters aimed to improve snatch performance through functional strength (strength-strength-balance) training [27]. The results showed a statistically significant improvement in both training and snatch performance. This supports the notion that functional strength training can positively impact specific athletic performance. Researchers have investigated the impact of periodic functional strength training on the functional movement screening (FMS) scores of high-risk sports college students [28]. Their findings revealed a significant improvement in the total FMS score in the experimental group after 12 weeks of following the functional strength training program (P < 0.05). Specifically, 45% of the volunteers who initially scored 14 on the FMS showed notable progress. These results align with previous studies, highlighting the superior effectiveness of functional strength training. Additionally, another study examined the effects of functional strength training on postural balance, gait, and functional strength in mentally-retarded teenagers [29]. The experimental group demonstrated a statistically significant increase (P < 0.05) in postural balance and muscle strength compared to the baseline, while the control group did not exhibit a similar improvement. Furthermore, a literature source recommended intelligent and personalized training that can be specifically tailored to individual training objectives [30]. Consequently, functional strength training has the potential to enhance participants’ strength in their core muscle groups, leading to improvements in strength, sensitivity, and balance.

In this work, the focus is on the analysis of kicking actions in football players. The analysis of football movement serves as a training aid, providing timely feedback to players when their kicking action deviates from the desired technique. Functional strength training has been a subject of investigation in relation to its impact on the effectiveness of football practice sessions. Previous research has explored the efficacy of functional strength training in injury prevention for adult male football players. The research has also examined the actual outcomes of implementing functional strength training when teaching football skills. The results have consistently demonstrated that functional strength training methods effectively enhance athletes’ abilities and performance, supporting its application in football training [31]. Additionally, academics have investigated the training and competition cycles of youth football players and highlighted the role of functional training in their physical preparation process. The integration of functional training into the workout plans of young athletes has been shown to be beneficial, enhancing their overall training outcomes [32]. The effects of functional training on lower limb strength in football players have also been explored in the literature. The findings indicate that functional training can improve lower limb strength and explosive power in football players. This underscores the importance of incorporating functional training into the physical training programs of football players, as it has the potential to contribute to their overall performance on the field [33]. The current study aligns with previous research, further supporting the notion that functional strength training can enhance athletes’ performance in the gym. The incorporation of functional training methods can significantly improve practice efficiency for adolescent football players, providing them with valuable tools to optimize their training and performance outcomes. By highlighting the existing literature and reinforcing the findings through this work, the benefits of functional strength training in the context of football training are further emphasized. This work conducted in this work contributes to the growing body of knowledge on functional training and its implications for football players, ultimately enhancing their overall athletic performance.

This article investigates the relationship between functional strength training and physical ability in soccer players. However, it is critical to acknowledge the limitations of this work, including the exclusion of speed training and the lack of clear conclusions regarding speed performance. Therefore, future research should consider incorporating speed training and expanding the sample size to provide a more comprehensive understanding of the developed program’s impact on improving the physical fitness of soccer players.

## 5. Conclusion

This work aims to investigate the relationship between functional strength training and speed and power in football players. The results indicate that functional strength training effectively increases strength and sensitivity in football players. However, this work did not find a significant correlation between functional strength training and players’ speed. These findings provide a theoretical basis for improving training methods and efficiency in teenage football players. However, there are limitations to consider. The small sample size used in this work may introduce potential biases and chance factors. Future research should expand the sample size to ensure more accurate results. Additionally, further investigation is needed to examine the impact of functional strength training on speed quality in football players. Exploring the link between motions and skills would also contribute to a more comprehensive understanding of the topic.

## Supporting information

S1 Data (XLSX)
